# Morphological identification of *Amphitetranychus* species (Acari: Tetranychidae) with crossbreeding, esterase zymograms and DNA barcode data

**DOI:** 10.1371/journal.pone.0221951

**Published:** 2019-09-13

**Authors:** Tea Arabuli, Mohamed Waleed Negm, Tomoko Matsuda, Yasuki Kitashima, Tea Abramishvili, Igor Andrijovych Akimov, Olga Valentynivna Zhovnerchuk, Sergei Yakovlevich Popov, Tetsuo Gotoh

**Affiliations:** 1 Laboratory of Applied Entomology and Zoology, Faculty of Agriculture, Ibaraki University, Ami, Ibaraki, Japan; 2 Institute of Zoology, Ilia State University, Kakutsa Cholokashvilli Ave, Tbilisi, Georgia; 3 Institute of Entomology, Agricultural University of Georgia, Kakha Bendukidze Campus, David Aghamashenebeli Alley, Tbilisi, Georgia; 4 Department of Plant Protection, Faculty of Agriculture, Assiut University, Assiut, Egypt; 5 Japan Society for the Promotion of Science, Chiyoda, Tokyo, Japan; 6 Department of Plant Pest Diagnostic, Laboratory of the Ministry of Agriculture, Vasil Godziashvili, Tbilisi, Georgia; 7 Schmalhausen Institute of Zoology, National Academy of Sciences of Ukraine, Vul. B. Khmelnytskogo, Kyiv, Ukraine; 8 Department of Plant Protection, Russian State Agrarian University—Moscow Timiryazev Agricultural Academy, Timiryazevskaya, Moscow, Russia; 9 Faculty of Economics, Ryutsu Keizai University, Ryugasaki, Ibaraki, Japan; Laboratoire de Biologie du Développement de Villefranche-sur-Mer, FRANCE

## Abstract

The genus *Amphitetranychus* Oudemans (Tetranychidae) consists of only three species, *A*. *quercivorus* (Ehara & Gotoh), *A*. *savenkoae* (Reck) and *A*. *viennensis* (Zacher). The original description of *A*. *savenkoae* was extremely simple and had no drawing of the aedeagus; however, a subsequent study described only the aedeagus. The present study investigated all three species in detail using a combination of morphological traits, crossbreeding experiments, esterase zymograms and the mitochondrial cytochrome *c* oxidase subunit I (*COI*) gene. Morphological differences in the peritremes and male aedeagi were observed among the three species. Complete reproductive isolation was confirmed in the reciprocal crosses between the morphologically similar *A*. *savenkoae* and *A*. *quercivorus* (no female offspring were produced). Esterase zymograms differed interspecifically, but not intraspecifically (among individuals in a given species). All three species formed clearly separate clades with 100% bootstrap values in the *COI* tree, and *A*. *savenkoae* was more closely related to *A*. *quercivorus* than to *A*. *viennensis*, which corresponded to the morphological similarity of their aedeagi and setal counts on tarsi IV. A key to *Amphitetranychus* species is provided.

## Introduction

Mites of the family Tetranychidae, also known as spider mites, are important pests of agricultural crops worldwide [1–3]. The genus *Amphitetranychus* is composed of only three species to date. This genus was established by Oudemans [4], assigning *A*. *viennensis* (Zacher) [5], from Germany, as the type species. In the same year, Hirst [6] described *Tetranychus crataegi* from England, which was later synonymized with *A*. *viennensis* [7]. The other two species are *A*. *savenkoae* (Reck) [8], which was described from Georgia [8–11] and Ukraine [12–14], and *A*. *quercivorus* (Ehara & Gotoh) [15], which was described from Japan [15] and Korea [16].

Conventionally, researchers use morphological characters to separate species within a genus [8, 12, 15, 17, 18]. Among the strains of *Amphitetranychus* species collected in the present study, *A*. *viennensis* was easily identified because of having a distinct aedeagal shape, whereas *A*. *quercivorus* and *A*. *savenkoae* were quite difficult to separate based only on the aedeagus. Because few morphological features are known to vary among *Amphitetranychus* species, they may be easier to be distinguished using genetic, physiological and biological methods [19, 20]. Therefore, we used a multidisciplinary approach to elucidate the taxonomy of these species based on: (i) morphological redescriptions with a key to species, (ii) crossbreeding experiments to assess the degree of reproductive isolation between *A*. *quercivorus* and *A*. *savenkoae* strains, (iii) esterase zymogram analysis, and (iv) sequencing of the partial mitochondrial cytochrome *c* oxidase subunit I (*COI*) gene and construction of a phylogenetic tree.

## Materials and methods

### Mite samples

A list of species and strains used in the present study is provided in Table 1. The species *A*. *savenkoae* was imported to Japan with the authorization of the Ministry of Agriculture, Forestry and Fisheries of Japan (no. 25-Y-532) on 29 Aug. 2013. Laboratory stocks were separately reared on leaf discs (ca. 16 cm^2^) of cherry, *Prunus* × *yedoensis* Matsum., for

**Table 1:** Collection data for experiments conducted on nine strains of three *Amphitetranychus* and two *Tetranychus* species examined in the present study.

Species	Locality	Geographic coordinates	Host plant	Date	# Voucher specimen^1^ (Experiment)^2^
***A*. *viennensis* (Zacher)**	Chiyoda, Tokyo, Japan	35°40'N–139°45'E	*Prunus armeniaca* L.	Sept. 21, 2010	613 (D, M)
	Chiyoda, Tokyo, Japan	35°40'N–139°45'E	*Prunus campanulata* Maxim.	June 12, 2012	885 (E, M)
	Chiyoda, Tokyo, Japan	35°40'N–139°45'E	*Prunus spachiana* (Lavall'e ex H. Otto)	June 12, 2012	886 (M)
	Ami, Ibaraki, Japan	36°02'N–140°12'E	*Prunus* x *yedoensis* Matsum.	Dec. 2, 2018	889 (D)
***A*. *quercivorus* (Ehara & Gotoh)**	Ishikari, Hokkaido, Japan	43°08'N–141°18'E	*Quercus mongolica* Fisch. ex Ledeb.	May 26, 2002	462 (D, E)
	Tsukuba, Ibaraki, Japan	36°06'N–140°06'E	*Q*. *mongolica*	July 9, 2003	610 (C, D, E, M)
***A*. *savenkoae* (Reck)**	Kherson, Ukraine	46°27'N–31°55'E	*Quercus robur* L.	Aug. 22, 2013	676 (C, D, E, M)
***T*. *urticae* (Koch) (Green form)**	Takikawa, Hokkaido, Japan	43°33'N–141°54'E	*Citrullus lanatus* (Thunb.)	July 16, 2003	181 (D)
***T*. *kanzawai* Kishida**	Kanaya, Shizuoka, Japan	34°48'N–138°23'E	*Camellia sinensis* (L.)	May 19, 1993	158 (D)

^1^Voucher specimens are preserved at the Laboratory of Applied Entomology and Zoology (Faculty of Agriculture, Ibaraki University) under the serial specimen numbers.

^2^Each strain of the five studied species was used for the following experiments: C: crossbreeding; D: DNA sequence; E: electrophoresis; M: morphology.

*A*. *viennensis*; deciduous oak, *Quercus mongolica* Fisch. ex Ledeb., for *A*. *quercivorus* and deciduous oak, *Q*. *robur* L., for *A*. *savenkoae*. The leaf discs were placed on water-saturated polyurethane mats in plastic dishes (90-mm diameter, 20-mm depth) at 25±1°C under a 16:8 h light:dark photoperiod. In winter, diapause females of *A*. *quercivorus* and *A*. *viennensis* were produced by the eggs, which were reared at 15°C under a 10:14 h light:dark photoperiod in the laboratory. When they reached adulthood, they were put in dark boxes at ca. 75% RH using saturated aqueous sodium chloride solution. The boxes were kept in a refrigerator at 5°C from December to April until 2013 after collection, the period when cherry and deciduous oak leaves were unavailable.

### Morphological analyses

Adult males and females for each species were mounted on permanent slides using Hoyer’s medium. The specimens were examined using an Olympus^®^ BX53 differential interference contrast microscope equipped with an Olympus^®^ DP72 digital camera and drawn by a camera lucida (U-DA, Olympus^®^) attached to the microscope. Illustrations were done with Adobe Illustrator (Adobe Systems Incorporated, USA). Measurements were performed using the imaging software Sensiv Measure^®^ ver. 2.6.0. All measurements are given in micrometers (μm) and correspond to mean values followed by the standard error (SE) and the minimum and maximum values (range) of specimens examined. Body length measurements represent the distance between tip of gnathosoma to end of idiosoma. Setae were measured from the center of the setal base to the end of the tip. Terminology and abbreviations used in the description follow Lindquist [21]. Leg setal counts are given in the order: coxa, trochanter, femur, genu, tibia and tarsus. Leg tactile and eupathidial setal numbers are provided first followed by solenidia in parentheses. Some of the voucher specimens of the redescribed species will be deposited in the collections of the Institute of Zoology, Ilia State University, Georgia, and others were deposited in the Laboratory of Applied Entomology and Zoology, Ibaraki University (AEZIU), Japan, under the serial voucher specimen numbers.

### Crossbreeding experiments

To determine the reproductive compatibility between *A*. *savenkoae* (Kherson, voucher specimen no. 676) and *A*. *quercivorus* (Tsukuba, no. 610), reciprocal crosses were carried out. Individual females in the teleiochrysalis stage (the final immature resting stage) that were obtained from each stock culture were transferred onto a small leaf disc (ca. 4 cm^2^) with two adult males obtained from the stock cultures of either the same or another species. Five days after the start of oviposition on each disc, the adults (female and males) were removed. Eggs on each leaf disc were checked daily to determine hatchability, survival rate of immature stages and ratio of female offspring. All experiments were carried out at 25°C and under a 16:8 h light:dark photoperiod.

### Electrophoresis

Electrophoresis (native polyacrylamide gel electrophoresis) was carried out to examine whether the three *Amphitetranychus* species could be discriminated by esterase zymograms. Live females were individually put into a 1.5-ml Eppendorf tube and then homogenized in 10 μl of 32% (w/v) sucrose with 0.1% Triton X-100 and 0.002% bromophenol blue by a pipette tip. The gels were 1-mm thick, 90-mm wide and 83-mm high, and contained Triton X-100 (concentration 0.05% in the separating gels and 0.1% in the stacking gels). The acrylamide concentration was 7.5% in the separating gels and 2.5% in the stacking gels. Electrophoresis was carried out at a constant current of 20 mA/gel at 5°C for 2 h. The electrode buffer was 25 mM Tris/192 mM glycine, pH 8.6. To detect non-specific esterase, the gels were incubated for 40 min at 30°C in 0.02% of α-naphthyl acetate in 0.1 M phosphate buffer (pH 6.5) which contained 1% acetone; then, the gels were soaked for 1 h in 0.2% Fast Blue BB salt solution. Ten individuals of each species were used for esterase zymogram analysis.

### Molecular analyses

We examined whether the three *Amphitetranychus* species could be distinguished by the mitochondrial *COI* gene sequences. Mite species were separately reared on leaf discs of each original host plant on a water-saturated polyurethane mat in a plastic dish (90-mm diameter, 20-mm depth) at 25ºC under a 16:8 h light:dark photoperiod, until analysis. Adult females from each species were arbitrarily selected and used for molecular analyses. Total DNA was extracted from the whole body of each female using PrepMan Ultra Reagent (Applied Biosystems, Foster City, CA). The primers C1-J-1718 [22] (5′-GGAGGATTTGGAAATTGATTAGTTCC-3′) and COI REVA [23] (5′-GATAAAACGTAATGAAAATGAGCTAC-3′) were used for polymerase chain reaction (PCR). PCR amplification was performed using ExTaq (Takara Bio, Shiga, Japan) under the following conditions: 3 min at 94°C; 35 cycles of 1 min at 94°C, 1 min at 45°C and 1.5 min at 72°C; and a final extension at 72°C for 10 min. The resultant DNA was purified using a MinElute PCR Purification Kit (Qiagen, Valencia, CA) and directly sequenced. Sequencing was carried out in both directions using the amplifying primers with a BigDye Terminator Cycle Sequencing Kit v.3.1 (Applied Biosystems) and on an ABI 3130xl automated sequencer. All obtained sequence data were deposited in DDBJ/EMBL/GenBank International Nucleotide Sequence databases under the accession numbers LC435686 (voucher specimen no. 610), LC435687 (no. 676), LC435688 (no. 676) and LC456179 (no. 889). The *COI* sequences for *A*. *quercivorus* (AB981238, no. 462), *A*. *viennensis* (AB981239, no. 613), *Tetranychus urticae* Koch (AB736076, no. 181) and *T*. *kanzawai* Kishida (AB736043, no. 158) were obtained from previously published data [24, 25]. Obtained sequences were aligned using CLUSTAL W in MEGA7 [26]. For the maximum likelihood (ML) analysis, we used the best-fit model (GTR+G model) chosen by MEGA7. ML trees were constructed with MEGA7. Branch robustness was tested by bootstrap analysis with 100 replications.

### Data analyses

One-way analysis of variance (ANOVA) was used to compare the influence of crosses on the number of eggs laid per female during the first five days of the oviposition period, egg hatchability, survival rate of immature stages and offspring sex ratio (i.e. the fraction of daughters). Means were compared among cross combinations using Tukey’s HSD test [27]. To normalize the data, log transformation was used for the number of eggs laid, and logistic transformation was used for egg hatchability, survival rate of immature stages and offspring sex ratio data [28].

## Results

### Morphology

Family Tetranychidae Donnadieu

Subfamily Tetranychinae Berlese

Tribe Tetranychini Reck

Genus *Amphitetranychus* Oudemans [4]

*Amphitetranychus* Oudemans [4]; Geijskes [29]; Pritchard & Baker [30]; Wainstein [10]; Mitrofanov et al. [12]; Ehara [31]. Type-species: *Tetranychus viennensis* Zacher [5]

Diagnosis. *Amphitetranychus* has no medio-dorsal spur on all empodia of the legs of both sexes; dorsocentral area of opisthosoma more or less with transverse striae; peritreme anastomosed distally; empodium usually without spur, splitting distally into three pairs of hairs; two sets of duplex setae of tarsus I well separated; with only one pair of para-anal setae; and tibia II with six tactile setae.

        *Amphitetranychus quercivorus* (Ehara & Gotoh) [15]

            [Japanese name: Mizunara-kudahadani]

                (Figs 1–3, Table 2)

**Fig 1:**
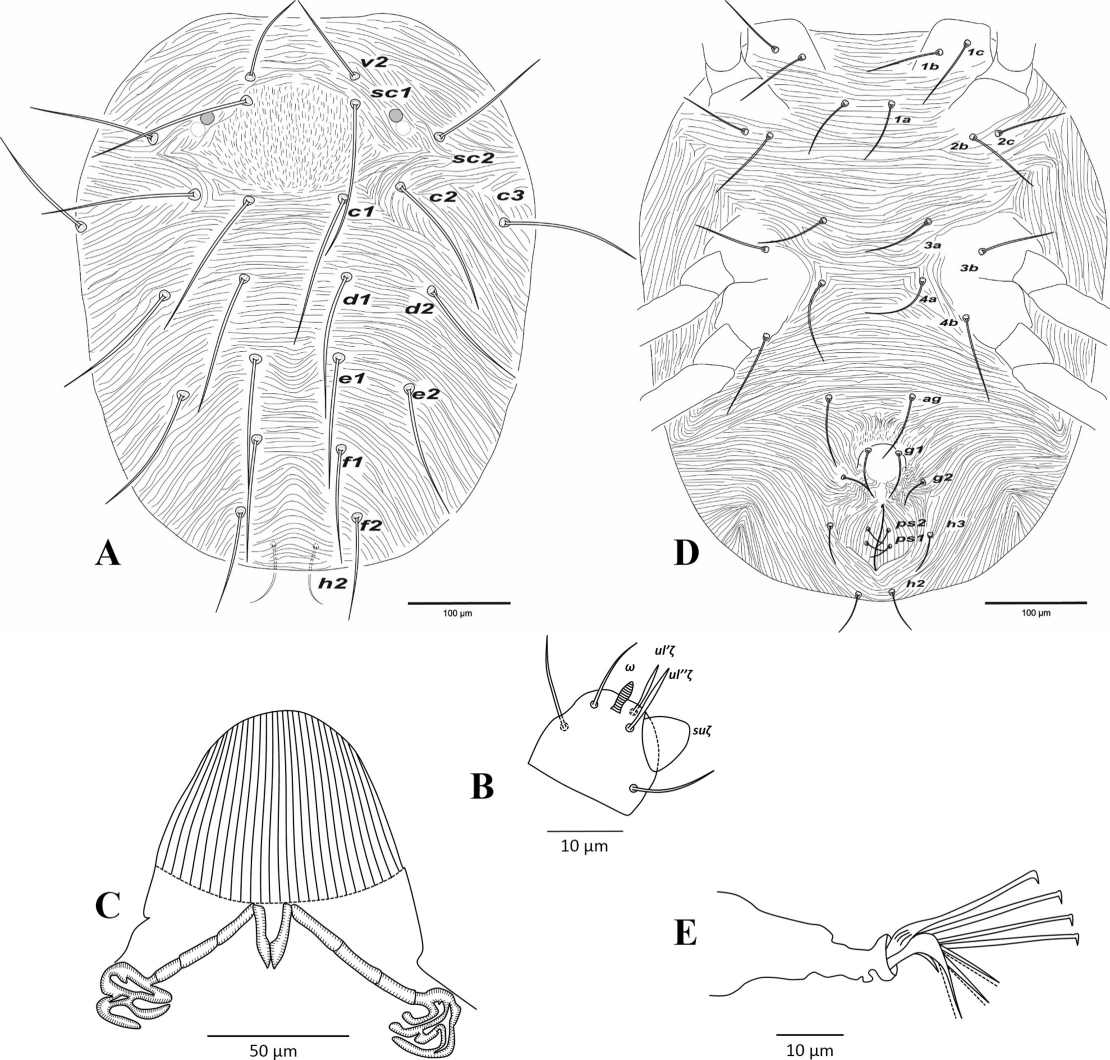
*Amphitetranychus quercivorus*, female—A. Dorsum, B. Distal segment of palpus, C. Peritremes, D. Venter, E. Empodium of leg I.

**Fig 2:**
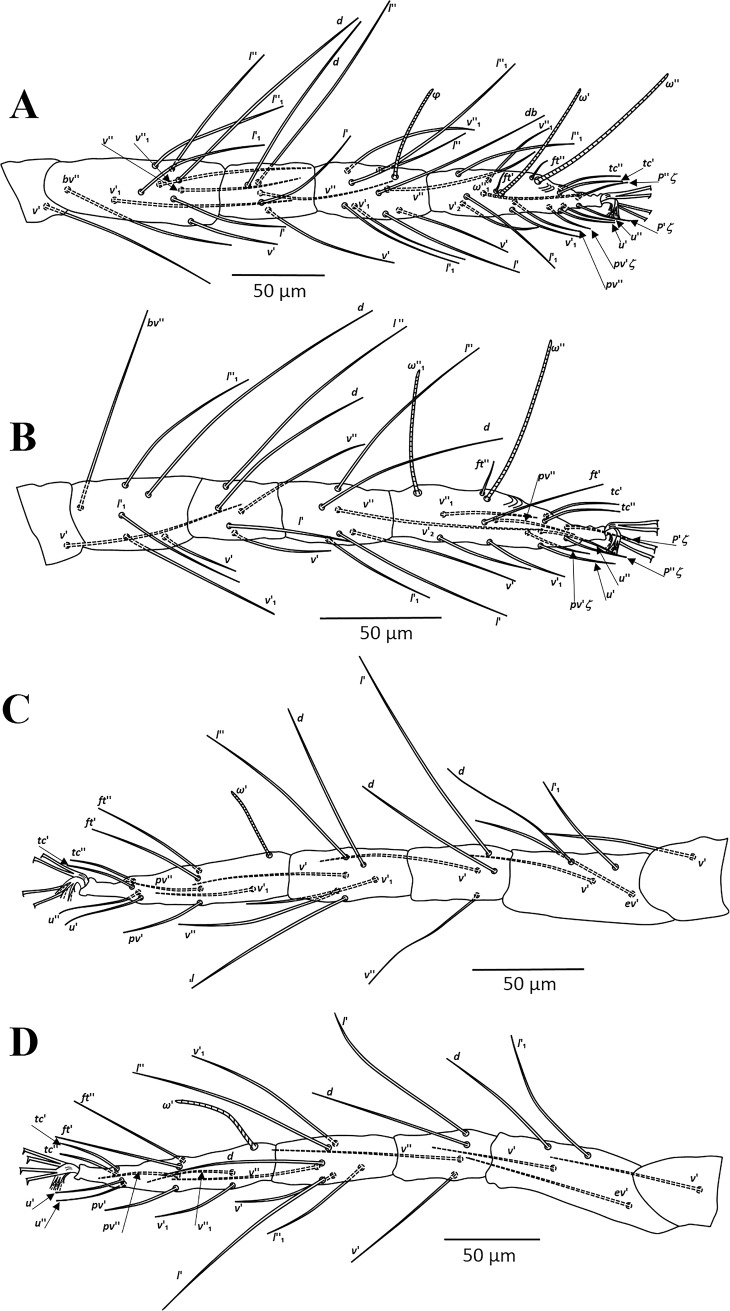
*Amphitetranychus quercivorus*, female—A. Leg I, B. Leg II, C. Leg III, D. Leg IV.

**Fig 3:**
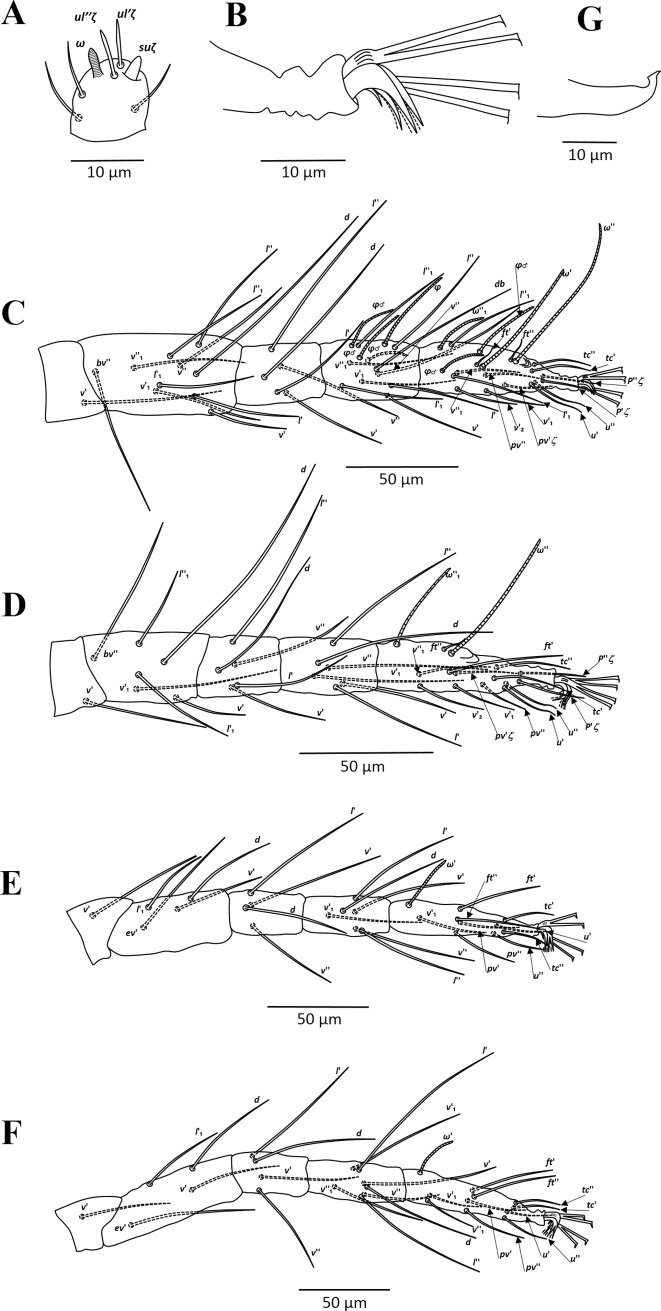
*Amphitetranychus quercivorus*, male—A. Distal segment of palpus, B. Empodium of leg I, C. Leg I, D. Leg II, E. Leg III, F. Leg IV, G. Aedeagus.

**Table 2:** Mean, standard error (SE) and range of taxonomic characters measured for females and males of *Amphitetranychus quercivorus*, *A*. *savenkoae* and *A*. *viennensis*.

Taxonomic characters	*A*. *quercivorus*						*A*. *savenkoae*						*A*. *viennensis*					
	Female (n = 10)			Male (n = 6)			Female (n = 10)			Male (n = 10)			Female (n = 10)			Male (n = 10)		
	Mean	SE	Range	Mean	SE	Range	Mean	SE	Range	Mean	SE	Range	Mean	SE	Range	Mean	SE	Range
**Body length**	623.6	7.3	590–668	423.33	2.2	417–433	629.1	4.9	609–651	441.7	3.2	426–455	614.6	10.3	567–648	450.7	2.6	440–461
**Gnathosoma**	136	1.4	130–145	105.16	2.3	97–114	133.8	1.1	129–139	109.8	0.5	107–112	115.2	1.4	110–122	88.7	1.8	80–95
**Body width**	396.1	5.5	375–429	221.33	2.8	213–232	385.6	1.8	379–392	237	3.5	215–253	398	13.2	341–444	249.1	2.1	240–259
**Dorsal setae**																		
***v2***	86.1	1.8	79–97	67.833	1.5	64–74	97.9	0.9	93–102	72.3	0.7	70–76	79.6	1.2	75–88	71.8	0.5	70–75
***sc1***	150.9	2	135–159	113.83	3.7	98–123	154.4	1.8	148–163	116.5	1	110–121	144	1.7	136–150	115.1	0.9	110–119
***sc2***	121.1	0.8	118–125	87.333	2.3	80–95	119.5	0.8	115–122	93.9	1.7	88–103	114.9	1.1	111–120	88.1	0.6	85–91
***c1***	136.3	1.4	130–143	105.16	1.6	101–111	141.6	1.2	137–148	106.1	0.9	103–110	132.3	1.3	127–137	104.4	0.9	100–110
***c2***	139.3	1.6	130–149	106.5	1.5	102–113	149.9	0.6	148–154	107.5	0.5	105–110	136.3	1.2	130–140	114.7	0.9	111–119
***c3***	136.4	1.8	129–147	100.33	1.9	95–108	138.8	0.6	136–141	102.4	1.4	98–109	126.5	1.2	123–135	98.4	1.9	92–106
***d1***	131.8	1.2	126–138	103.33	2.2	98–111	144.1	1.1	140–150	100.7	1.2	96–107	126.3	1.5	120–132	99.4	0.5	97–102
***d2***	135.5	1.2	129–140	103	0.9	100–107	140.5	0.5	138–142	103.6	1	98–107	124.1	0.8	120–127	104.4	0.9	100–107
***e1***	123.8	1	120–128	92	1.1	90–97	132.4	0.6	130–135	94.5	0.6	93–97	119.6	0.9	116–123	89.8	0.9	85–94
***e2***	130.9	1.4	124–137	96.33	1.2	93–102	140.4	0.9	138–145	97.8	2.1	91–107	124.5	0.5	123–127	92.5	0.6	90–95
***f1***	112.4	1.2	107–119	72.5	2.4	63–79	129.5	1.4	123–135	69.8	0.4	68–73	109.8	0.8	106–113	78.8	0.9	75–83
***f2***	101.3	1.5	95–108	36.5	1.5	32–41	108.5	0.8	105–111	39.3	0.5	37–41	96.4	2.6	86–107	33.9	0.3	32–35
***h2***	48	0.8	45–53	24.66	1	21–28	57.7	1.2	52–63	27.6	0.7	24–30	49.1	0.6	45–51	23	0.5	21–25
**Palpal setae**																		
***suζ* length**	6.19	0.1	6–6.4	3	0.1	2.5–3.4	6.91	0.1	6.5–7.2	3.51	0.1	3.1–3.9	5.77	0	5.6–6	3.54	0.1	3.2–3.8
***suζ* width**	6.66	0.2	6–7.2	2.53	0.2	2.1–3	5.14	0.1	4.8–5.4	2.31	0.1	2.1–2.7	6.34	0.2	5.4–6.9	2.41	0	2.3–2.5
***ω***	4.14	0	4–4.3	3.86	0.1	3.6–4.2	3.81	0	3.6–4	3.74	0.1	3–4.2	4.55	0.1	4.1–5.1	4.7	0.1	4.2–5.1
***ul'ζ***	5.94	0.1	5.5–6.4	5.56	0.1	5–5.9	5.89	0.1	5.4–6.5	4.68	0.1	4.1–5.1	6.22	0	6.1–6.3	5.71	0.1	5.1–6.2
***ul'' ζ***	7.38	0.2	6.7–8.6	6.88	0.1	6.7–7.2	8.6	0.1	8.1–9.3	7.47	0.1	7–8	7.84	0.1	7.5–8.2	6.98	0.1	6.7–7.2
**Leg I**	366.4	3.8	346–382	269.16	3.6	258–281	391.2	6.2	355–415	292.5	3.1	280–305	373.2	5.1	351–400	285.6	2.5	270–296
**Leg II**	278.9	2.9	267–291	225.66	2.8	218–236	290.9	2.7	280–303	235.4	1.3	230–241	268.3	2.4	259–279	237.7	1.8	230–245
**Leg III**	286.1	1.9	279–296	244.16	3.2	232–255	319.1	3.1	301–335	248.3	2.9	238–264	267.9	3.5	250–286	244.8	1.7	235–251
**Leg IV**	328.4	3.9	311–345	286.5	4.1	273–301	364.9	1.1	360–370	286.4	1.5	280–292	330.3	5.6	301–352	287.3	4.1	270–309
**Femur I**	87.2	1.9	81–100	78.66	1.6	73–83	106.9	1.6	100–113	81.7	0.9	78–88	100.9	1.5	95–111	76.7	0.9	73–80
**Genu I**	53.1	0.7	49–56	39.5	0.8	36–42	58.4	0.7	54–61	45.5	0.5	43–48	50.4	0.8	47–55	43.9	0.8	40–47
**Tibia I**	59.2	0.8	55–63	43	0.6	41–45	63.7	0.9	60–68	45.4	0.6	43–48	57.7	0.8	54–61	48.7	0.6	46–51
**Tarsus I**	102	2.4	89–109	83	0.9	79–85	114.7	1.3	109–121	84.8	1.5	78–91	98.5	0.9	95–103	78	1.1	74–82
**SDD**^**1**^ **TsI**	89.3	1.5	80–95	71.83	1	69–75	95.1	1.5	90–101	76.7	0.3	75–78	90.3	0.4	88–92	72.7	0.5	70–75
**SPD**^**2**^ **TsI**	68.7	1.4	62–75	54.66	1.5	49–60	65.9	1.3	60–71	50	1.1	46–55	69.2	0.3	68–71	49.6	0.5	47–52
**TDD**^**3**^ **TsI**	7.9	0.5	6–11	7.83	0.5	7–10	12.8	0.3	11–14	8.3	0.4	7–10	8.9	0.2	8–10	7.4	0.2	7–8
**TPD**^**4**^ **TsI**	8.5	0.5	6–10	8.5	0.7	7–11	11.4	0.2	10–12	8.8	0.4	7–10	8.3	0.2	7–9	7.4	0.2	7–8
**SD TsII**	65.3	1.5	58–74	53.33	0.9	50–56	66.4	1.4	61–73	55.1	0.7	52–57	69.2	0.8	66–72	59.3	0.3	58–61
**TD TsII**	7.4	0.4	6–9	7.33	0.3	6–8	10.1	0.3	9–11	7.4	0.3	6–9	7.5	0.2	7–8	6.5	0.2	6–7
**Aedeagus**																		
**Shaft length**^**5**^	-	-	-	12.16	0.1	11.9–12.4	-	-	-	9.43	0.1	9.1–9.6	-	-	-	9.95	0.1	9.5–10.3
**Shaft width**	-	-	-	5.18	0.2	4.4–5.9	-	-	-	5.69	0.1	5.4–5.9	-	-	-	5.61	0.2	4.9–6.5
**Knob length**	-	-	-	2.2	0.1	2–2.4	-	-	-	2.18	0	2.1–2.3	-	-	-	8.03	0.1	7.6–8.7

^1^Solenidion of distal duplex seta;

^2^solenidion of proximal duplex seta;

^3^tactile seta of distal duplex seta;

^4^tactile seta of proximal duplex seta;

^5^dorsal margin of shaft.

Description. Female (n = 10). Body reddish. Dorsum—Dorsal body setae slender, longer than distances between bases of consecutive setae, not set on tubercles. Prodorsum with scattered longitudinal striation in the area between setae (*v2*, *sc1* & *c1*); hysterosomal striations transverse medially and oblique laterally, striae slightly curved between setae *e1* and convex at area between setae *f1* and *f2* (Fig 1A). Gnathosoma—Palptarsus with three tactile setae, spinneret (*suζ*), spindle shaped solenidion (*ω*) and a pair of eupathidia (*ul’ζ* and *ul”ζ*) (Fig 1B). Stylophore rounded anteriorly, with longitudinal striation dorsally; peritreme anastomosed distally (Fig 1C). Venter—Pregenital area with vague longitudinal striae. One pair of aggenital setae (*ag*), two pairs of genital setae (*g1*, *g2*), one pair of paranal setae (*h3*) and two pairs of pseudanal setae (*ps1*, *ps2*) present (Fig 1D). Legs—Empodia I–IV split into three pairs of ventrally directed hairs (Fig 1E). Leg chaetotaxy as follows (solenidia in parenthesis; Fig 2A–2D):

leg I 2-1-10-5-9(1)-13+(1)+2 duplexes
leg II 2-1-6-5-6-12+(1)+1 duplex
leg III 1-1-4-4-6-9+(1)
leg IV 1-1-4-4-7-10+(1)

Tarsus I with four tactile setae and one solenidion proximal to proximal set of duplex seta (Fig 2A). Tarsus II with three tactile setae and one solenidion proximal to duplex seta, one tactile seta (*ft’*) near level of duplex seta (Fig 2B).

Male (n = 6). Body pale greenish-yellow. Dorsum—Dorsal body setae simple, acicular, longer than distances between bases of consecutive setae. Gnathosoma—Palptarsus as in Fig 3A; peritreme as in female. Legs—Empodia as in Fig 3B. Leg setal counts as follows (Fig 3C–3F):

leg I 2-1-10-5-9+(4)-13+(3)+2 duplexes
leg II 2-1-6-5-6-12+(1)+1 duplex
leg III 1-1-4-4-6-9+(1)
leg IV 1-1-4-4-7-10+(1)

Tarsus I with four tactile setae and three solenidia proximal to proximal set of duplex seta, one solenidion (*φ♂*) near duplex seta (Fig 3C). Aedeagus—Shaft of aedeagus with dorsal margin upturned distally forming a neck which ends with a terminal knob, constructing an approximately 45-degree angle between the axis of the knob and dorsal margin of shaft (Fig 3G).

Materials examined. Ten females and six males (voucher specimen no. 610), Tsukuba, Ibaraki, Japan (36°06′N–140°06′E, Y. Kitashima leg.), on *Quercus mongolica* (Fagaceae) (see Table 1).

        *Amphitetranychus savenkoae* (Reck) [8]

            (Figs 4–6, Table 2)

**Fig 4:**
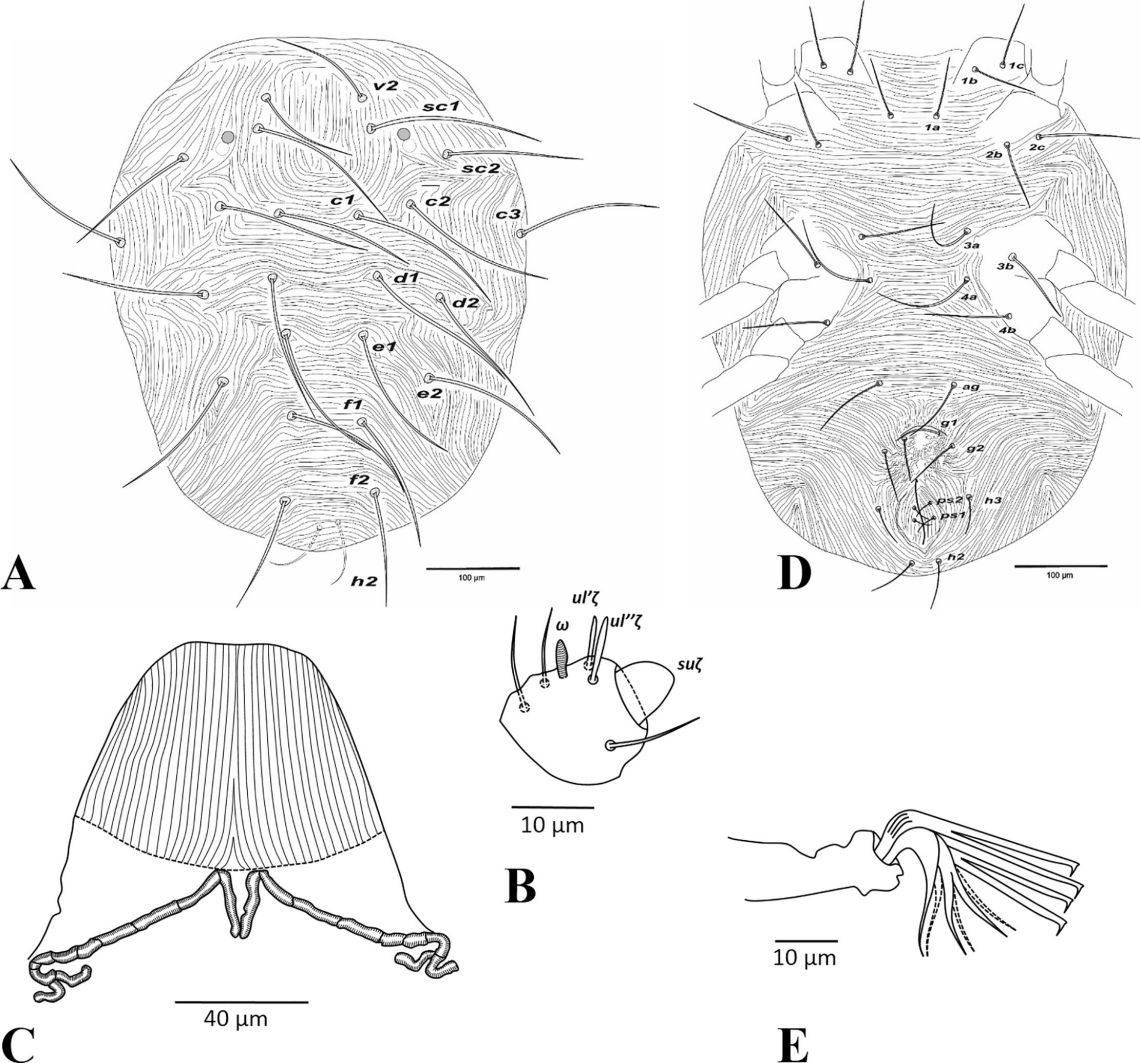
*Amphitetranychus savenkoae*, female—A. Dorsum, B. Distal segment of palpus, C. Peritremes, D. Venter, E. Empodium of leg I.

**Fig 5:**
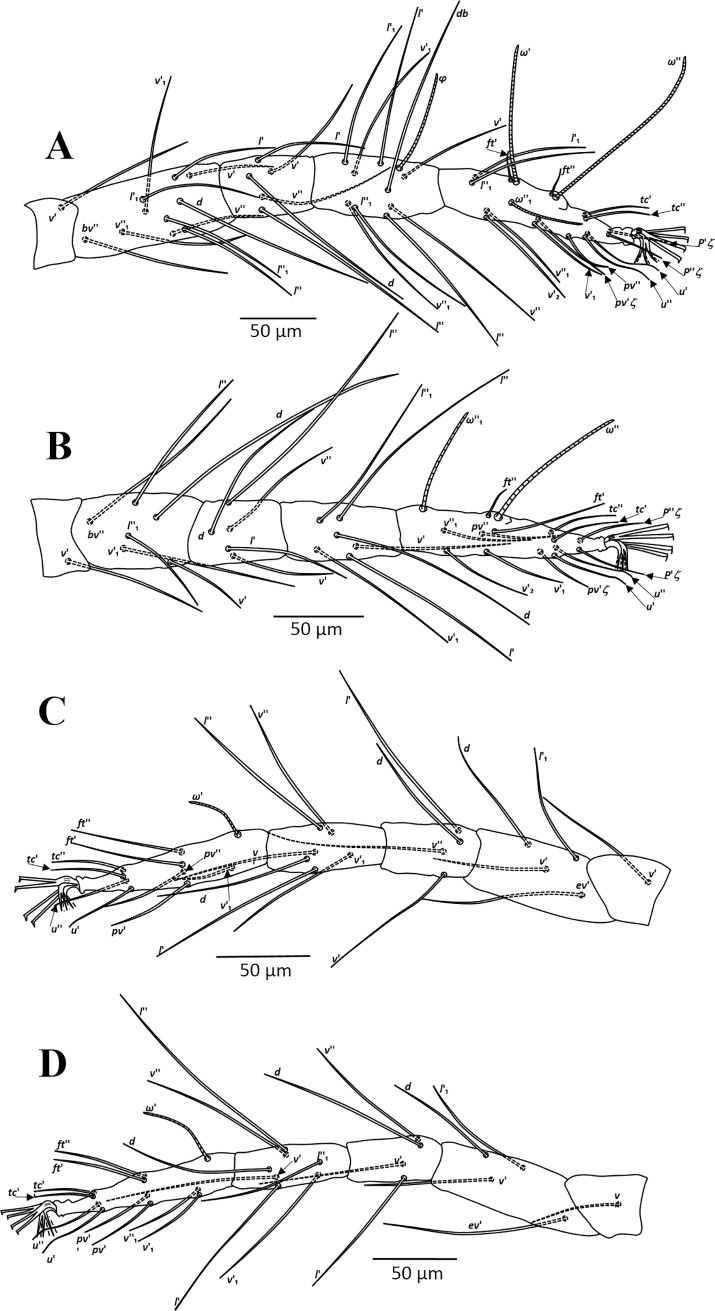
*Amphitetranychus savenkoae*, female—A. Leg I, B. Leg II, C. Leg III, D. Leg IV.

**Fig 6:**
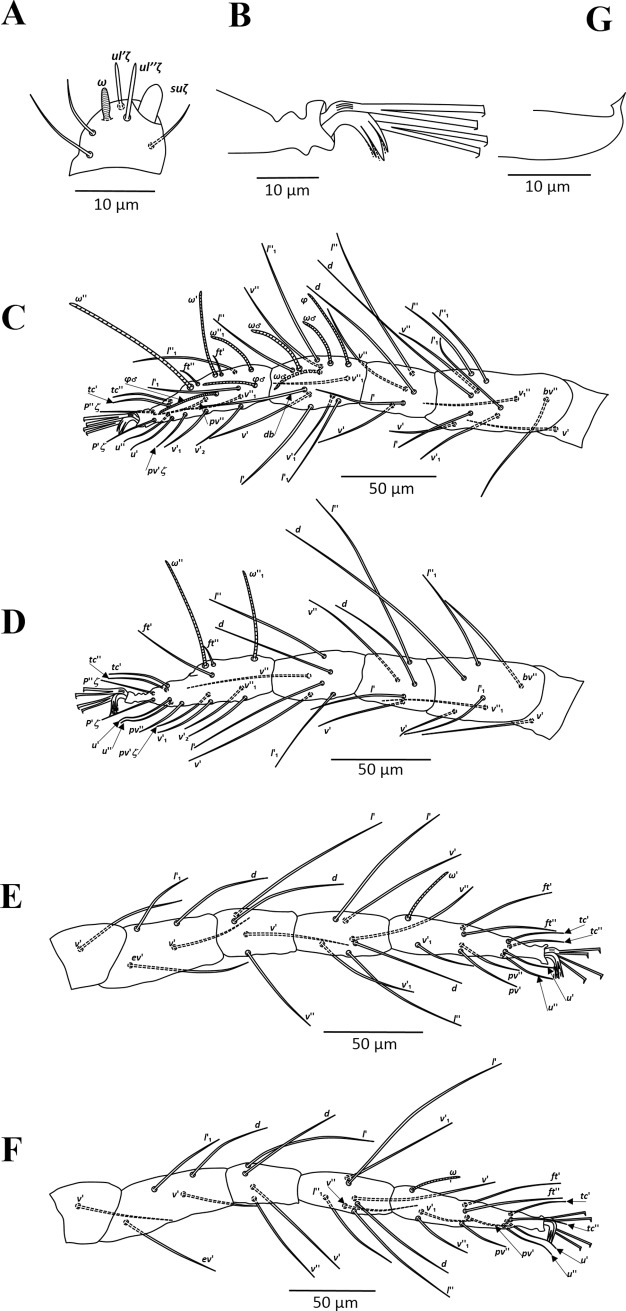
*Amphitetranychus savenkoae*, male—A. Distal segment of palpus, B. Empodium of leg I, C. Leg I, D. Leg II, E. Leg III, F. Leg IV, G. Aedeagus.

Description. Female (n = 10). Body reddish. Dorsum—As in *A*. *quercivorus*, dorsal body setae slender, longer than distances between bases of consecutive setae, not set on tubercles. Prodorsum with distinct longitudinal striations centrally; hysterosomal striations transverse medially, oblique and longitudinal laterally (Fig 4A). Gnathosoma—Palptarsus as in Fig 4B. Stylophore normal in shape; peritreme bifurcated distally (Fig 4C). Venter—Pregenital area with conspicuous longitudinal striae. Aggenital, genital, paranal and pseudanal setae at normal positions (Fig 4D). Legs—Empodia I–IV split into three pairs of hairs, with proximal pair claw-like (Fig 4E). Leg setal counts as in *A*. *quercivorus* (Fig 5A–5D). Tarsus I with four tactile setae and one solenidion proximal to proximal set of duplex seta, with solenidion (*ω”*_*1*_) adjacent to duplex seta (Fig 5A). Tarsus II with five tactile setae and one solenidion proximal to duplex seta (Fig 5B).

Male (n = 10). Body pale greenish-yellow. Dorsum—Dorsal setae slender, longer than distances between bases of consecutive setae. Gnathosoma—Palptarsus as in Fig 6A; peritreme as in female. Legs—Empodia as in Fig 6B. Leg setal counts as in *A*. *quercivorus* (Fig 6C–6F). Tarsus I with four tactile setae and two solenidia proximal to proximal set of duplex seta (Fig 6C). Tarsus II with five tactile setae and one solenidion proximal to duplex seta, two tactile setae at the same level of duplex seta (Fig 6D). Aedeagus—Similar in shape to *A*. *quercivorus*; however, aedeagal knob markedly wider than neck (Fig 6G).

Materials examined. Ten females and 10 males (voucher specimen no. 676), Kherson, Ukraine (46°27′N–31°55′E, T. Gotoh leg.), on *Quercus robur* (see Table 1).

*Amphitetranychus viennensis* (Zacher) [5]

[Japanese name: Ôtô-hadani]

(Figs 7−9, Table 2)

**Fig 7:**
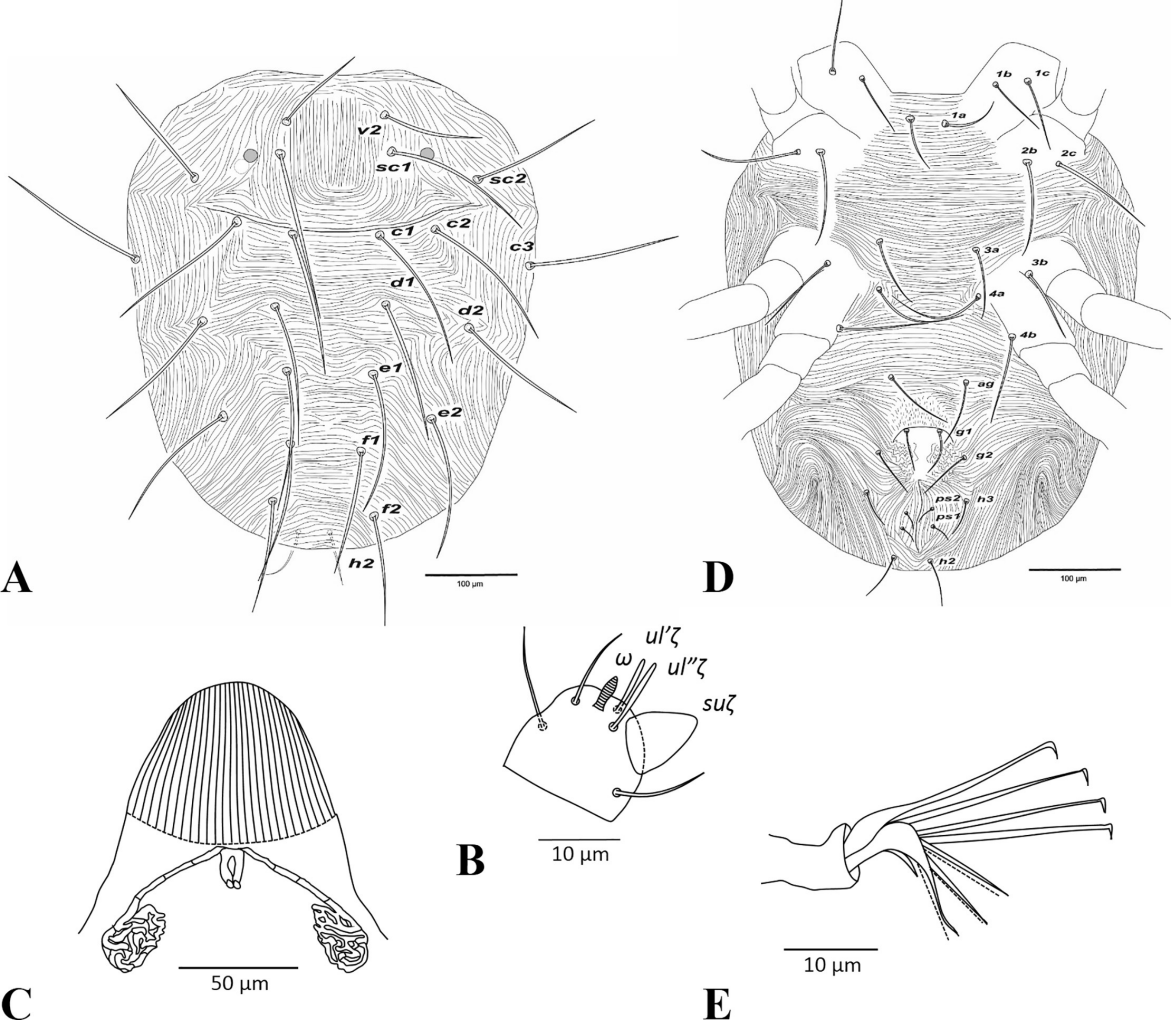
*Amphitetranychus viennensis*, female—A. Dorsum, B. Distal segment of palpus, C. Peritremes, D. Venter, E. Empodium of leg I.

**Fig 8:**
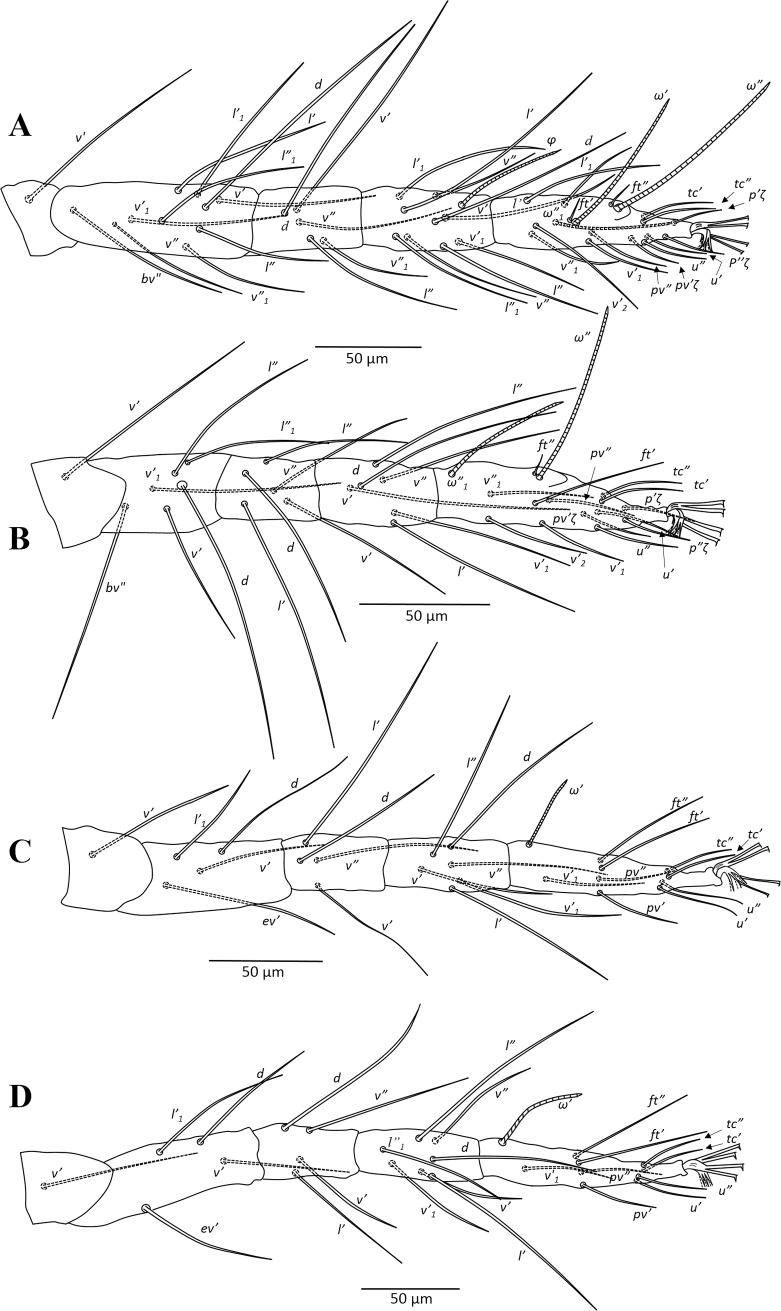
*Amphitetranychus viennensis*, female—A. Leg I, B. Leg II, C. Leg III, D. Leg IV.

**Fig 9:**
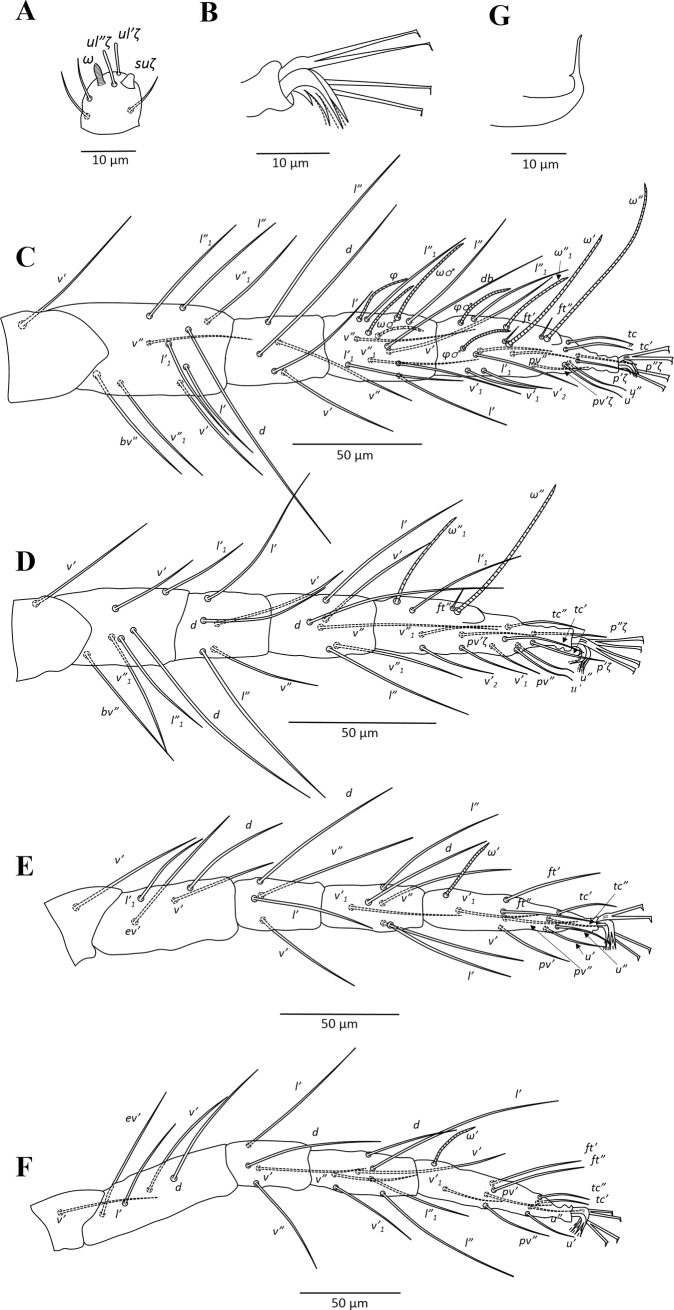
*Amphitetranychus viennensis*, male—A. Distal segment of palpus, B. Empodium of leg I, C. Leg I, D. Leg II, E. Leg III, F. Leg IV, G. Aedeagus.

Description. Female (n = 10). Body reddish with whitish legs. Dorsum—Dorsal body setae long, not set on tubercles. Prodorsum with longitudinal striations; hysterosomal striations transverse medially, oblique and longitudinal laterally (Fig 7A). Gnathosoma—Palptarsus as in Fig 7B. Stylophore rounded anteriorly with longitudinal striation dorsally; peritreme densely anastomosed distally (Fig 7C). Venter—Pregenital area with visible speckles connected to longitudinal striae towards genital flap. Genital and anal setae present at normal positions (Fig 7D). Legs—Empodia I−IV split into three pairs of hairs, with proximal pair claw-like (Fig 7E). Leg setal counts as follows (Fig 8A–8D):

leg I 2-1-10-5-9(1)-13+(1)+2 duplexes
leg II 2-1-6-5-6-12+(1)+1 duplex
leg III 1-1-4-4-6-9+(1)
leg IV 1-1-4-4-7-9+(1)

Tarsus I with four tactile setae and one solenidion proximal to proximal set of duplex seta (Fig 8A). Tarsus II with four tactile setae and one solenidion proximal to duplex seta, two tactile setae near the level of duplex set (Fig 8B).

Male (n = 10). Body dark greenish with pale red prodorsum. Dorsum—Dorsal body setae slender, longer than distances between bases of consecutive setae. Gnathosoma—Palptarsus as in Fig 9A; peritreme as in female. Legs—Empodia as in Fig 9B. Leg setal counts as follows (Fig 9C–9F):

leg I 2-1-10-5-9+(4)-13+(3)+2 duplexes
leg II 2-1-6-5-6-12+(1)+1 duplex
leg III 1-1-4-4-6-9+(1)
leg IV 1-1-4-4-7-9+(1)

Tarsus I with five tactile setae and three solenidia proximal to proximal set of duplex seta, one solenidion (*ω”*_*1*_) adjacent to duplex seta (Fig 9C). Tarsus II with three tactile setae and one solenidion proximal to duplex seta (Fig 9D). Aedeagus—Shaft of aedeagus gradually narrowing posteriorly towards the neck, upturned distally forming a tapering knob which has a minute projection anteriorly and blunt tip posteriorly (Fig 9G).

Materials examined. Five females and five males (voucher specimen no. 613), Chiyoda, Tokyo, Japan (35°40′N–139°45′E, T. Gotoh leg.), on *Prunus armeniaca* (Rosaceae); three females and three males (voucher specimen no. 885), Chiyoda, Tokyo, Japan (35°40′N–139°45′E, T. Gotoh leg.), on *P*. *campanulata*; two females and three males (voucher specimen no. 886), Chiyoda, Tokyo, Japan (35°40′N–139°45′E, T. Gotoh leg.), on *P*. *spachiana* (see Table 1).

### Key to species of *Amphitetranychus*

1. Female: Peritremes densely anastomosed distally (Fig 7C). Male: aedeagal knob long, extending posteriorly to a blunt tip (Fig 9G). Tarsus IV with 10 setae (nine tactile and one solenidion) in both females and males (Figs 8D & 9F). . . . . . . . . . . . . . . . . . . . . . . . . . . . . . . . . . . . . . . . . . . . . . . .*viennensis*
- Female: Peritremes less anastomosed. Male: aedeagal knob distinctly shorter, with posterior projection gently pointed. Tarsus IV with 11 setae (10 tactile and one solenidion) in both females and males (Figs 2D, 3F, 5D & 6F). . . . . . . . . . . . . .2
2. Female: Peritremes bifurcate distally (Fig 4C). Male: aedeagal knob markedly wider than neck (Fig 6G). . . . . . . . . . . . . . . . . . . . . . . . . . . . . . . . . . . . . . . . . .*savenkoae*
- Female: Peritremes otherwise, not bifurcated, with anastomosed grooves (Fig 1C). Male: aedeagal knob slightly wider than neck (Fig 3G). . . . . . . . . . . . . . . . . . . . . . . . . . . . . . . . . . . . . . . . . . . . . . . . . . . . . .*quercivorus*

### Crossbreeding experiments

In the intra-specific crosses, total egg production, hatchability, survival rate of immature stages and offspring sex ratio (% females) were not significantly different in both *A*. *quercivorus* and *A*. *savenkoae* (Table 3). The reciprocal crosses between *A*. *quercivorus* and *A*. *savenkoae* produced no female offspring (Table 3), indicating that these two species are reproductively isolated. In arrhenotokous species such as spider mites, unfertilized eggs develop into haploid males, whereas fertilized eggs develop into diploid females. Reduction in number of eggs laid and lower egg hatchability and survival rate of immature stages were observed in the inter-specific crosses compared with the intra-specific crosses.

**Table 3:** Number of eggs laid during the first five days of the oviposition period, egg hatchability, survival rate of immature stages and sex ratio of F1 progeny from crosses between *Amphitetranychus quercivorus* (Tsukuba) and *A*. *savenkoae* (Kherson) at 25°C under a 16:8 h light:dark photoperiod.

Cross			*N*^1^	Total number of eggs per female		Hatchability (%)		Survival rate (%) of immatures		% Female	
Female	×	Male									
*A*. *quercivorus*		*A*. *quercivorus*	22	13.27±0.35	a	98.71±0.60	a	95.02±1.11	a	79.81±1.17	a
*A*. *savenkoae*		*A*. *savenkoae*	16	15.25±0.87	a	97.71±1.10	a	95.20±1.32	a	81.59±0.97	a
*A*. *quercivorus*		*A*. *savenkoae*	14	12.86±0.55	a	76.45±6.97	b	82.36±2.57	b	0.00±0.00	b
*A*. *savenkoae*		*A*. *quercivorus*	18	14.72±0.69	a	86.45±4.08	b	89.79±1.77	ab	0.00±0.00	b
*F*_3,66_^2^				2.810*		7.708***		6.845***		3262.528***	

^1^Number of pairs tested.

^2^Data are shown as mean±S.E. Means differed significantly at *P*<0.05 (*) and *P*<0.001 (***) (ANOVA). Values in a column followed by the same letters are not significantly different at the 5% level (Tukey HSD test).

### Esterase zymograms

**Fig 10:**
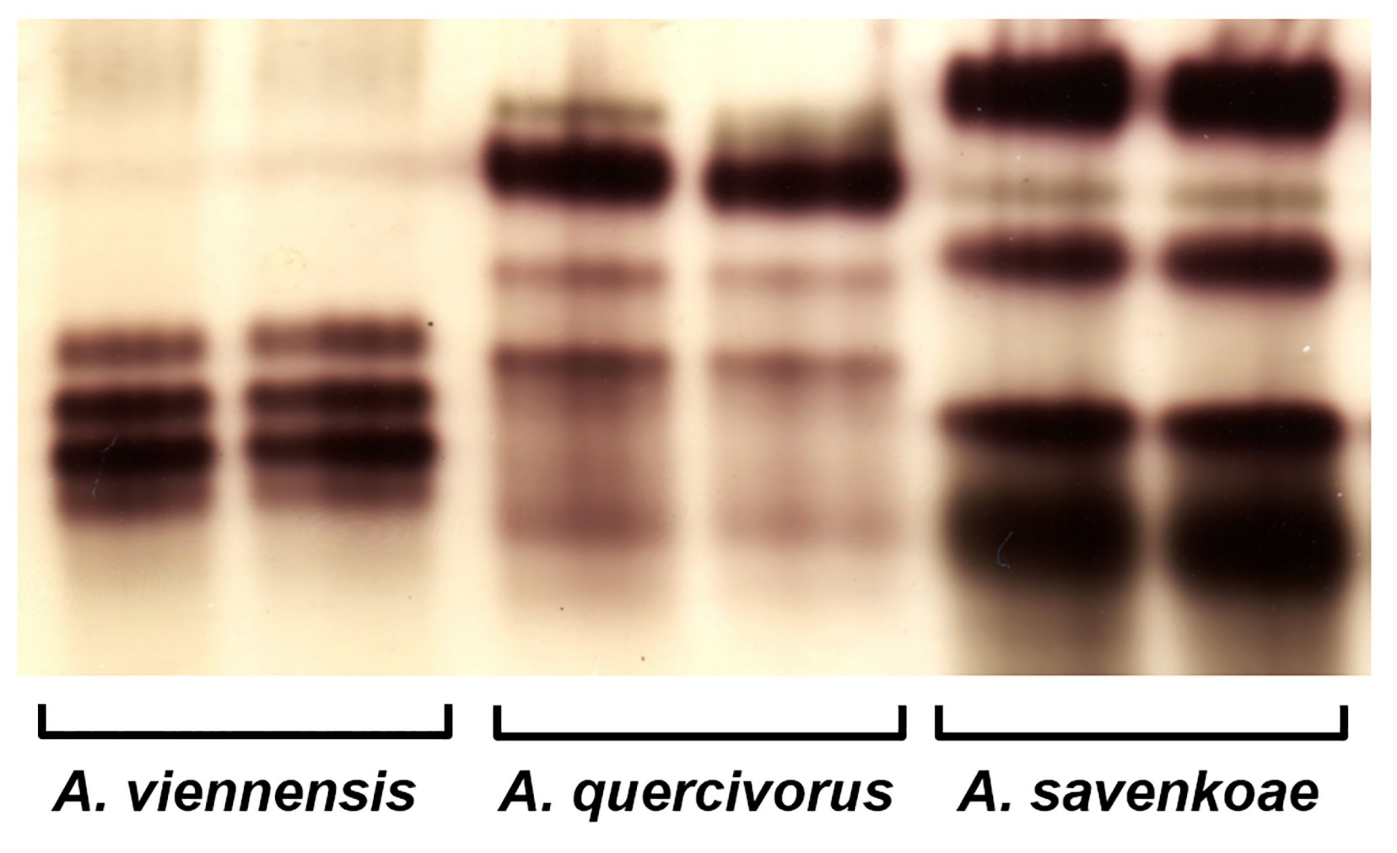
Esterase zymograms of *A*. *viennensis* (Chiyoda, voucher specimen no. 885), *A*. *quercivorus* (Tsukuba, no. 610) and *A*. *savenkoae* (Kherson, no. 676).

There was no intra-specific variation in esterase zymograms for the three *Amphitetranychus* species. Esterase band patterns were species-specific and the three *Amphitetranychus* species could be electrophoretically discriminated (Fig 10).

### Molecular analyses

**Fig 11:**
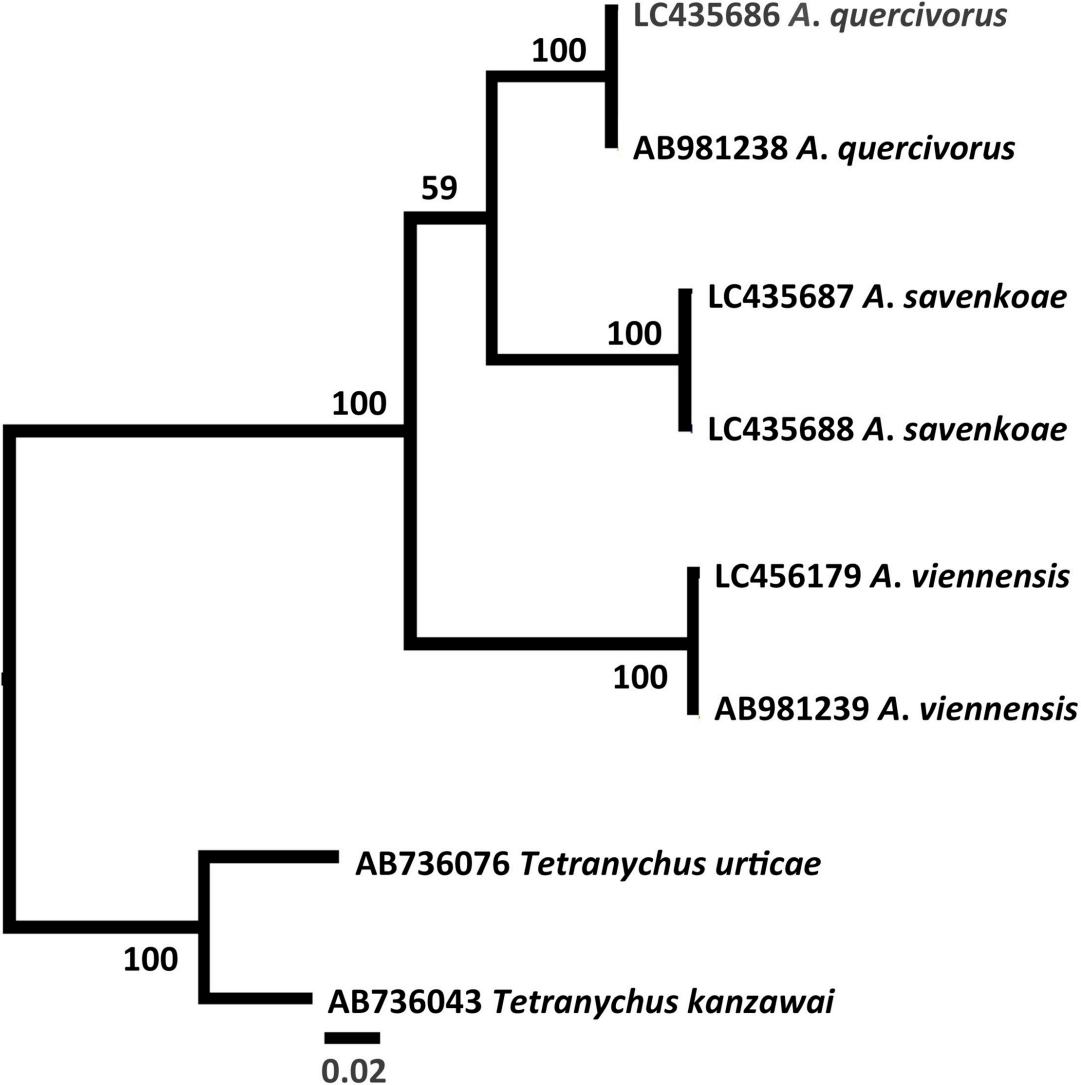
Maximum likelihood tree based on 618 bp of the mitochondrial *COI* gene of the genus *Amphitetranychus* using the GTR+G model. Bootstrap values based on 100 replications are indicated at the nodes. Only bootstrap values >50% are shown. Each operational taxonomic unit is indicated by accession number and scientific name.

After alignment, the *COI* fragment had 618 nucleotide sites, of which 113 were parsimony-informative and contained no insertions or deletions. In the *COI* tree (Fig 11), all three *Amphitetranychus* species formed clearly separate clades with 100% bootstrap values. The genetic distances between species were 7–8% between *A*. *quercivorus* and *A*. *savenkoae*, 9–10% between *A*. *quercivorus* and *A*. *viennensis*, and 11–12% between *A*. *savenkoae* and *A*. *viennensis*. These results indicated that the *COI* sequences are effective for identifying *Amphitetranychus* species. Additionally, although the associated bootstrap value was relatively low (59%), *A*. *savenkoae* was more closely related to *A*. *quercivorus* than *A*. *viennensis*.

## Discussion

The present work provides morphological, crossbreeding and molecular evidence that the genus *Amphitetranychus* currently includes three taxonomically distinct species: *A*. *quercivorus*, *A*. *savenkoae* and *A*. *viennensis*. Here, we discuss each piece of evidence based on different geographic strains of these species.

Comparative examination of morphological features for the five examined strains (one Ukrainian and four Japanese) showed a clear separation between the taxonomic identities of the three species based on female peritremes and male aedeagi. This separation was consistent and similar to that observed in other strains from Korea [16], except for *A*. *savenkoae*, which is redescribed in detail for the first time here. *Amphitetranychus savenkoae* was first described by Reck [8] from specimens collected on *Quercus* sp. (Fagaceae) in Georgia and was subsequently recorded from Ukraine [12, 13]. Reck’s [8] original description of *A*. *savenkoae* was not sufficiently detailed and lacked drawings and information on the male aedeagus. Additionally, although Mitrofanov et al. [12] provided a drawing of the male aedeagus, they did not provide any descriptions. Moreover, Reck [8] described female tarsi I and II with 18 and 15 setae, respectively; however, the present specimens had tarsi I and II with only 16 and 14 setae, respectively. This variation in setal counts may be because the old Russian acarologists did not separate in their descriptions between tactile setae and solenidia. We conclude that the three species have strong morphological similarities in measurements and leg chaetotaxy, but the most distinguishable trait was the shape of the peritreme.

The crossbreeding experiments confirmed that the studied strains of *A*. *quercivorus* and *A*. *savenkoae* were reproductively isolated and represent different biological species. Concerning *A*. *viennensis*, Gotoh and Takayama [32] reported its reproductive incompatibility with *A*. *quercivorus* by crossing populations collected from rosaceous and fagaceous trees, respectively. Therefore, the cross experiments herein were conducted only between the two species, *A*. *quercivorus* and *A*. *savenkoae*. Furthermore, *A*. *viennensis* can be easily separated from the other two species by its distinct shape of aedeagus.

Furthermore, the band patterns of the esterase zymograms were clearly different among the three *Amphitetranychus* species. Enohara and Amano [33] and Gotoh et al. [34] also applied the same technique to separate various *Tetranychus* species, and considered enzyme zymograms to be a powerful tool for species separation.

The *COI* nucleotide sequences also showed that the three *Amphitetranychus* species are different species because they formed separate clades. Khaing et al. [16] reported 11% variation in *COI* sequences between the Korean strains of *A*. *quercivorus* and *A*. *viennensis*. The *COI* variation (9–10%) between the Japanese strains of *A*. *quercivorus* and *A*. *viennensis* in our experiments was also similar to the *COI* variation between the Korean strains.

We conducted various analyses that all produced the same results, which clarified the taxonomic status of the three *Amphitetranychus* species. The aedeagal shapes indicate the existence of two different morphological groups, which is consistent with the higher similarity between *A*. *quercivorus* and *A*. *savenkoae*. This close relationship is also supported by the *COI* analysis, which showed that *A*. *quercivorus* is more closely related to *A*. *savenkoae* than to *A*. *viennensis*. Recently, several case studies have used integrative taxonomy and employed the same methods to separate cryptic species [23, 35, 36]. We conclude that integration of different approaches is required to distinguish different and closely related taxa and, therefore, to delimit species.
